# Preclinical evaluation of ^68^Ga-radiolabeled trimeric affibody for PDGFRβ-targeting PET imaging of hepatocellular carcinoma

**DOI:** 10.1007/s00259-023-06260-x

**Published:** 2023-05-31

**Authors:** Huawei Cai, Zhao Li, Qiuxiao Shi, Hao Yang, Liu Xiao, Mufeng Li, Hua Lin, Xiaoai Wu, Tianshan She, Lihong Chen, Lin Li, Xiaofeng Lu

**Affiliations:** 1 Department of Nuclear Medicine & Laboratory of Clinical Nuclear Medicine, West China Hospital, Sichuan University, Chengdu, 610041 China; 2 NHC Key Lab of Transplant Engineering and Immunology, West China Hospital, Sichuan University, Chengdu, 610041 China; 3 Institutes for Systems Genetics, Frontiers Science Center for Disease-Related Molecular Network, West China Hospital, Sichuan University, Chengdu, 610041 China; 4 Sichuan Provincial Engineering Laboratory of Pathology in Clinical Application, West China Hospital, Sichuan University, Chengdu, 610041 China; 5 Department of Biochemistry and Molecular Biology, West China School of Basic Medical Sciences & Forensic Medicine, Sichuan University, Chengdu, 610041 China

**Keywords:** Hepatocellular carcinoma, PDGFRβ, Affibody, ^68^Ga, PET

## Abstract

**Purpose:**

Hepatocellular carcinoma (HCC) is a highly vascularized solid carcinoma and tumor vessel–targeted molecular imaging might be effective for early diagnosis of HCC. Herein, we developed a novel trimeric affibody (Z_TRI_) with highly specific binding to the platelet-derived growth factor receptor beta (PDGFRβ). The aim of this study is to evaluate the feasibility of ^68^Ga-radiolabeled Z_TRI_ ([^68^Ga]Ga-DOTA-Z_TRI_) as PET tracer for diagnosis of HCC.

**Methods:**

The bioinformatics analysis of clinical database and immunoblotting of clinical specimens were performed to validate the potential of PDGFRβ as HCC biomarker. The trimeric affibody Z_TRI_ was conjugated with DOTA-NHS-ester and radiolabeled with ^68^Ga to produce [^68^Ga]Ga-DOTA-Z_TRI_ conjugate. Immunoreactivity and specific uptake of [^68^Ga]Ga-DOTA-Z_TRI_ were assessed by dose-dependent cell binding, autoradiography, and biodistribution analysis. [^68^Ga]Ga-DOTA-Z_TRI_ PET/CT scanning of diethylnitrosamine (DEN)-induced primary HCC rats and a rare case of idiopathical HCC rhesus monkey was performed to evaluate the imaging capability and radiation dosimetry of [^68^Ga]Ga-DOTA-Z_TRI_ in vivo.

**Results:**

Excessive PDGFRβ was validated as a representative biomarker of HCC neovascularization. The radiolabeling of [^68^Ga]Ga-DOTA-Z_TRI_ was achieved at more than 95% radiochemical yield. In vitro assays showed specific uptake of [^68^Ga]Ga-DOTA-Z_TRI_ in HCC tumor vessels by autoradiography. Animal PET/CT imaging with [^68^Ga]Ga-DOTA-Z_TRI_ successfully visualized the tumor lesions in primary HCC rats and rhesus monkey, and indicated radiation absorbed dose of 2.03E-02 mSv/MBq for each scanning.

**Conclusions:**

Our results demonstrated that [^68^Ga]Ga-DOTA-Z_TRI_ conjugate could be applied as a promising PET tracer for early diagnosis of hepatocellular carcinoma.

**Supplementary information:**

The online version contains supplementary material available at 10.1007/s00259-023-06260-x.

## Introduction

Hepatocellular carcinoma (HCC) has currently been the second leading cause of cancer-related death worldwide (1). The average 5-year survival rate of HCC is less than 20% since 70 to 90% of patients tend to be diagnosed at advanced stages (2). Accurate early diagnosis is effective in improving the survival of patients and now is a matter of urgent need in clinic. Although positron emission tomography (PET) with ^18^F-fluorodeoxyglucose ([^18^F]-FDG) was helpful in detection of a variety of malignancies (3), this technique is not recommended for the detection of HCC due to its limited sensitivity (4, 5). [^11^C]-Acetate, choline derivatives, fibroblast activating protein (FAP), and prostate-specific membrane antigen (PSMA)–targeted derivatives have been developed as distinguished PET tracers (6, 10). Nevertheless, further studies on novel biomarkers are needed to better define the characterization of HCC, with particular regard to the dual-tracer PET imaging modality.

Tumoral angiogenesis has been identified as a prerequisite of solid tumors, and therefore increasing attention has been given to antiangiogenic therapy and tumor vessel–targeted diagnosis (11, 12). Several endothelium-associated biomarkers including vascular endothelial growth factor/receptor (VEGF/VEGFR) and integrin αvβ3 have been evaluated for tumor imaging (13, 14). In addition to endothelium cells, pericytes are the other major mural cells of tumor vessels and plays a crucial role in regulating tumor microvascular morphogenesis (15), suggesting that it is a distinguished tumor vascular target (16). Platelet-derived growth factor receptor β (PDGFRβ) is the representative biomarker on the surface of tumor-associated pericytes (17). Consequently, several PDGFRβ-specific probes were developed for imaging of mice bearing subcutaneous xenografts of glioblastoma (18), breast cancer (19), and colorectal carcinoma (20). In our previous studies, a PDGFRβ-specific dimeric affibody (Z_PDGFRβ_) was developed and successfully radiolabeled with zirconium-89 (^89^Zr). PET imaging of mice bearing PDGFRβ^+^ colorectal xenografts with ^89^Zr produced high-contrast tumor images with clean liver background (21), which greatly triggered our interest to evaluate the potential of PDGFRβ-targeted imaging for HCC diagnosis. In addition, as the vascularization of subcutaneous tumor xenografts is different to that of the primary tumor grafts, we attempted to evaluate the PDGFRβ-targeted tracer in animals with primary HCC. Moreover, to produce a radioactive tracer with further greater affinity for PDGFRβ, we produced a trimerized PDGFRβ-specific affibody (designated as Z_TRI_) and radiolabeled it with ^68^Ga to prepare a novel radioactive conjugate, [^68^Ga]Ga-DOTA-Z_TRI_. Finally, the specific uptake and the imaging capability of [^68^Ga]Ga-DOTA-Z_TRI_ for primary HCC lesions in both rat and rhesus monkey were evaluated.

## Materials and methods

### Production and characterization of Z_TRI_ affibody

A self-trimerizing domain (TRI) derived from collagen XV (22) was genetically fused to the C terminus of Z_PDGFRβ_ to produce trimeric PDGFRβ affibody (Z_TRI_). Z_TRI_ is obtained through *Escherichia coli* M15 system according to our previous descriptions (23). The purity and molecular weight of the Z_TRI_ were evaluated by sodium dodecyl sulfate–polyacrylamide gel electrophoresis (SDS-PAGE) or size-exclusion chromatography (SEC) performed on a Superdex G-75 Increase 10/30 column (GE Healthcare, Anaheim, CA).

Biolayer interferometry performed on the Octet platform (Pall ForteBio LLC, CA) was used to measure the affinity of the Z_TRI_ for PDGFRβ. The recombinant human PDGFRβ-Fc protein (Sino Biological, Beijing, China) were immobilized on a protein A-coated biosensor followed by insertion into solutions containing different concentrations (0.5–30 μM) of Z_TRI_ for association. The affinity constant (K_D_) was calculated according to a 1:1 binding model according to our previous study (24).

### *Preparation of [*^*68*^*Ga]Ga-DOTA-Z*_*TRI*_

Radiolabeling of Z_TRI_ was achieved by bifunctional chelator 1,4,7,10-tetraazacyclododecane-1,4,7,10-tetraacetic acid mono-*N*-hydroxysuccinimide ester (DOTA-NHS-ester; Macrocyclics, TX).

Briefly, Z_TRI_ was mixed with DOTA-NHS-ester at a molar ratio of 1:5 (protein to DOTA) in sodium bicarbonate solution (pH 8.0–8.5) at room temperature for 4–6 h to produce [^68^Ga]Ga-DOTA-Z_TRI_. Unconjugated DOTA-NHS-ester was removed by ultrafiltration centrifuge tube with cut-off molecule of 2000 (Millipore, CA). Conjugation of DOTA to Z_TRI_ was verified using Q Exactive Orbitrap mass spectrometry. ^68^Ga was obtained from an IGG-100 ^68^Ge/^68^Ga generator (Eckert & Zieglert Isotope Inc, Germany) by eluting with 0.1 M ultrapure hydrochloric acid. For radiolabeling, the DOTA-Z_TRI_ solution (50 μg conjugate in 50 μl deionized water) was mixed with [^68^Ga]GaCl_3_-HCl eluent (1 MBq per microgram of protein) and pH adjusted to 3.5–4.0 by the addition of 1 M sodium acetate. After incubation at 50 °C for 20 min, a small fraction of mixture was measured by radio-instant thin-layer chromatography (radio-ITLC) to determine the radiolabeling efficacy. The radio-ITLC was performed with Waterman No.1 paper as solid phase and 1 M citric acid as mobile phase. The [^68^Ga]Ga-DOTA-Z_TRI_ product Rf = 0–0.2, while free ^68^Ga ion Rf = 0.8–1.0.

### Flow cytometry and western blotting

Human vascular pericytes were purchased from ScienCell (CA, USA) and cultured in specific media at 37 °C in a 5% CO_2_ humidified atmosphere. To measure the cell binding capability, 3 × 10^5^ cells in 100 μl PBS were incubated with FAM-labeled Z_TRI_ at 4 °C for 1.5 h and then analyzed on a Cytomics FC500 (Backman, CA, USA). The expression of PDGFRβ in pericyte was also measured by flow cytometry with PE anti-human PDGFRβ (AF385, R&D, MN; Clone:18A2).

For immunoblotting, HCC tissues were extracted using lysis buffer (Beyotime, Shanghai, China) and separated by SDS-PAGE. Western blotting was performed with antibody anti-PDGFRβ and horseradish peroxidase (HRP)–conjugated donkey antigoat IgG (ZenBio, Chengdu, China). GAPDH was used as loading control.

### Immunofluorescence and autoradiography assay

To detect the colocalization of PDGFRβ and Z_TRI_ in tumor vessels, HCC tissues were sectioned as 6-μm slices under frozen conditions, followed by incubation with goat anti-human PDGFRβ (AF385; R&D, MN), rabbit anti-rat PDGFRβ (380,772; ZenBio, Chengdu, China), or rat anti-human CD31 (303,102, BioLegend, CA; Clone:WM59) at 37 °C for 1.5 h. Subsequently, the tissues were washed with PBS and incubated with corresponding secondary antibodies (BioLegend, CA) for an additional 0.5 h prior to observation under a Zeiss LSM800 laser scanning confocal microscope (Zeiss, Germany). Similar procedures were performed to detect the cellular distribution of Z_TRI_ in the tissues.

For autoradiography (ARG), tissues were sectioned into 15-μm slices under frozen conditions followed by 4% paraformaldehyde fixing. After incubation with [^68^Ga]Ga-DOTA-Z_TRI_ (0.74 MBq in PBS) for 0.5 h, the tissues were washed and placed on a phosphor screen cassette (GE Healthcare, MA) in the dark for 4 h. The imaging films were scanned at a pixel size of 20 × 20 μm using an Amersham Typhoon imaging system (GE Healthcare).

### Animal models

All the animal experiments were performed in compliance with protocol approved by the Animal Care and Use Committees guidelines in West China Hospital and Sichuan University (2018189A for rodent animals and 2020248A for rhesus monkey). To induce the primary HCC, Wistar rats (male, 150–200 g, 6 weeks) were fed with diethylnitrosamine (DEN) according to our previous descriptions (25). A rare case of naturally idiopathic HCC rhesus monkey (8 years old, male, 5.85 kg) and another healthy male monkey (11 years old, 5.77 kg) were included in this study. Tissue samples from animals were prepared as 10-µm-thick slices for pathological analysis of hematoxylin & eosin (H&E) or alpha-fetoprotein (AFP) immunohistochemical staining.

### Biodistribution and biosafety

Biodistribution of [^68^Ga]Ga-DOTA-Z_TRI_ was investigated in BALB/c mice (16–18 g, 4–6 weeks old, *N* = 5). Mice were intravenously injected with 740 kBq of tracer and sacrificed at 5 min, 15 min, 0.5 h, 1 h, 2 h, and 4 h post-injection. The organs were harvested and radioactivity was measured by a Wizard 2480 gamma counter (PerkinElmer).

To evaluate the acute toxicity, [^68^Ga]Ga-DOTA-Z_TRI_ (50 MBq) or an equivalent Z_TRI_ (50 μg) was intravenously injected into BALB/c mice (*N* = 5), followed by measuring the body weight every day for 7 days. At the end of the observation, liver and kidney of mice were collected for H&E staining.

### PET imaging

Rats were intravenously injected with 5.55 MBq of [^68^Ga]Ga-DOTA-Z_TRI_ and scanned at 1 h post-injection using an IRIS small animal PET/CT imaging system (Inviscan SAS, Strasbourg, France). Subsequently, tissues were harvested for autoradiography. For monkey imaging, [^68^Ga]Ga-DOTA-Z_TRI_ was administered with a dose of 3.7 MBq/kg by intravenous bolus injection. After 1 h post-injection, PET images were obtained over 15 min using a Discovery PET/CT 710C (GE Healthcare). [^18^F]-FDG scanning was performed by GE Signa PET/MR with similar procedure.

PET images were analyzed using Osirix MD software version 10.0.4 (Pixmeo SARL), and data were expressed as standard uptake values (SUV) in animals. Specific organ regions of interest were drawn by PMOD version 4.004 and radiation absorbed dose in monkey was calculated by OLINDA/EXM version 2.2.3 (Hermes Medical Solutions).

### Bioinformatics analysis and clinical specimen collection

The expression of angiogenesis-associated biomarkers in HCC were first accessed by bioinformatics analysis. RNA-seq data of 371 HCC samples and 226 normal samples were obtained from The Cancer Genome Atlas (TCGA) database and the Genotype-Tissue Expression (GTEx) database, respectively. The expression levels of angiogenesis-associated genes in HCC and normal tissues were analyzed and compared using Gene Expression Profiling Interactive Analysis (GEPIA, http://gepia.cancer-pku.cn/), a web-based tool to deliver fast and customizable functionalities based on TCGA and GTEx data (26). In addition, datasets GSE14323 comprising 41 cirrhotic tissues derived from patients with HCV infections and 19 normal liver tissues were also analyzed to detect the relative expression of major angiogenesis-associated genes in HCC tissues.

Clinical specimens included paracancerous tissues derived from HCC patients and sectioned residual tissues of the donor liver for transplantation. The cryopreserved tissues were obtained from the Biobank of West China Hospital under the guide of the Ethics Committee of the Institute.

### Statistics

Data were analyzed using Prism 8 version 8.1.0 (GraphPad). Comparisons were made using a two-tailed, Mann–Whitney–Wilcoxon test or ANOVA followed by Bonferroni’s multiple-comparison test. *P* values < 0.05 were considered statistically significant.

## Results

### ZTRI shows highly specific binding to PDGFRβ in HCC

**Fig. 1:**
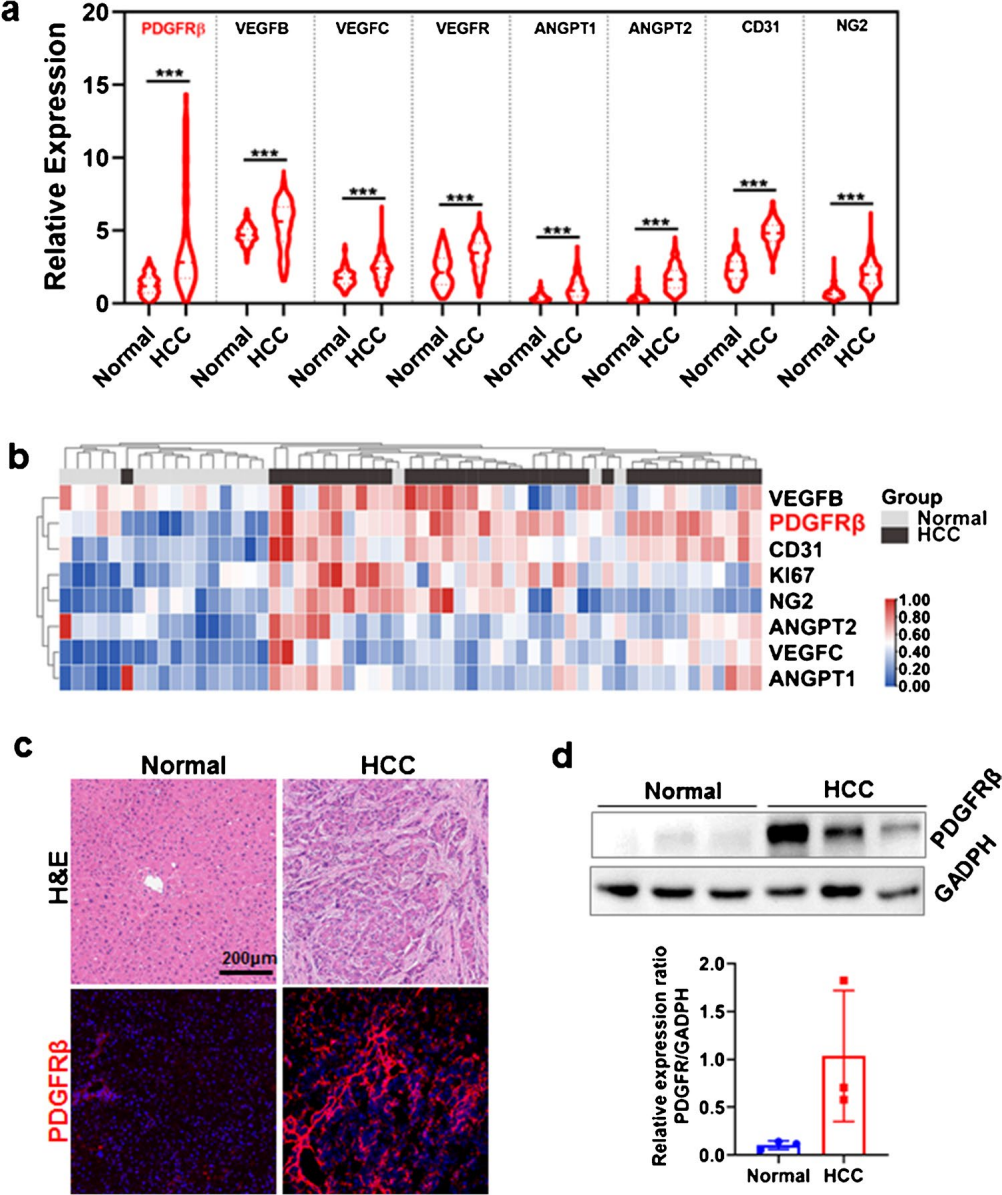
**a** The transcriptomic profile of typically upregulated angiogenesis-associated genes in tumor tissues derived from HCC patients. **b** Protein expression profiles of angiogenesis-associated genes that were correlated with the severity of HCC. **c** H&E and PDGFRβ staining of liver tissues derived from HCC patients or residual normal liver tissues of the donor liver for transplantation. PDGFRβ-positive cells (red) were visualized by antibodies against human PDGFRβ. The nuclei of the cells were stained by DAPI (blue). **d** Western blot of PDGFRβ in normal or HCC tissues (*n* = 3)

A series of typical angiogenesis-associated genes including VEGFB, VEGFC, VEGFR, ANGPT1, ANGPT2, CD31, NG2, and PDGFRβ were analyzed by GEPIA to assess the gene transcription and expression in public HCC patients’ database. As shown in Fig. 1 a and b, transcriptomics data and protein expression profiles of these genes indicated a strong correlation to HCC, and PDGFRβ is one of the most hyperactive genes. Furthermore, individual specimens obtained from our institutional biobank also verified more than tenfold higher expression of PDGFRβ in HCC tumor tissues than that in healthy liver tissues by immunofluorescence (Fig. 1c) and Western blotting (Fig. 1d). These results indicate that PDGFRβ might be a promising biomarker for vessel-targeted molecular imaging of HCC.

**Fig. 2:**
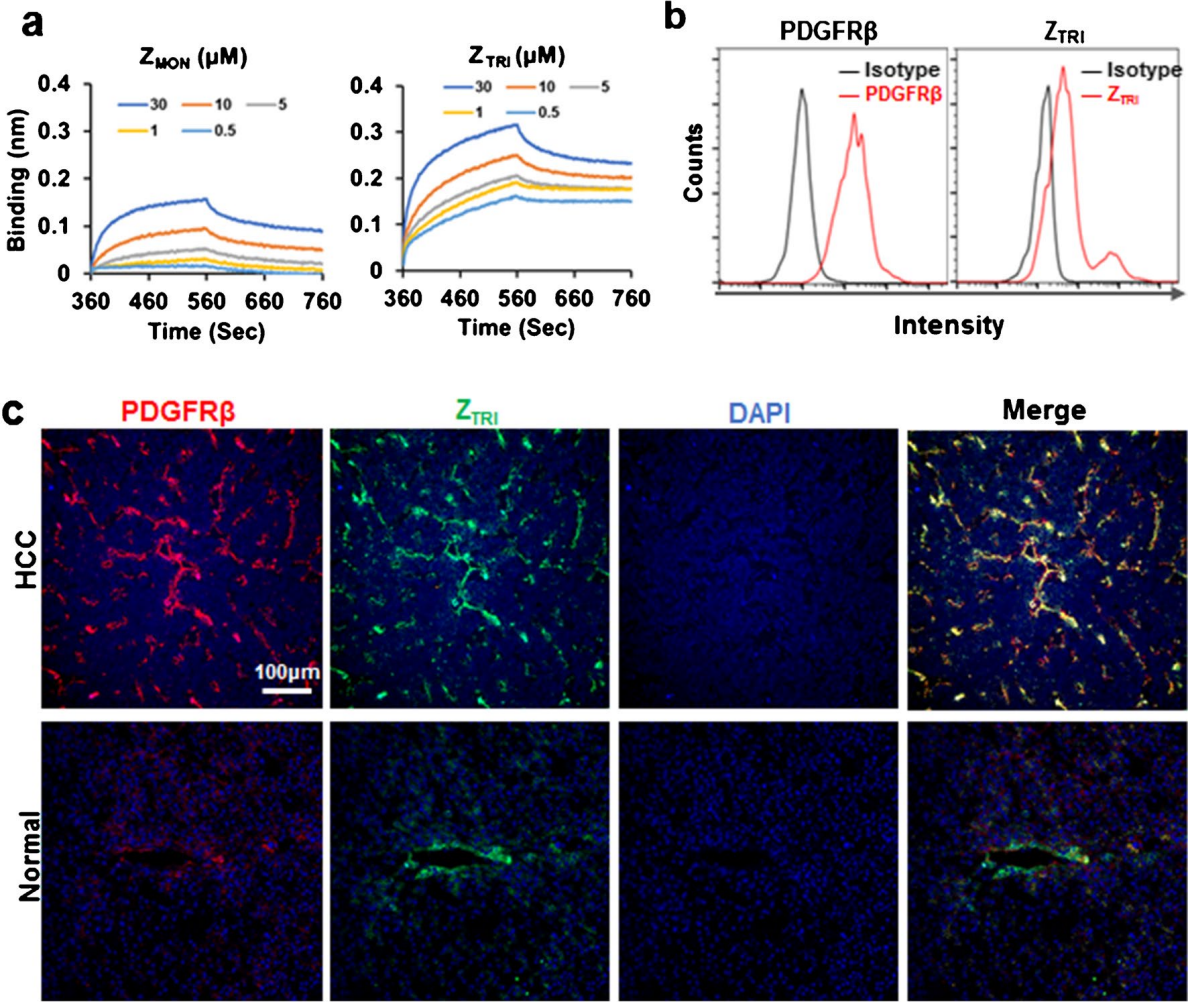
Z_TRI_ shows high affinity for PDGFRβ. **a** Affinity of Z_MON_ and Z_TR_ affibody to PDGFRβ analyzed by biolayer interferometry at different concentrations. **b** Binding of Z_TRI_ to PDGFRβ.^+^ pericyte was evaluated by flow cytometry. **c** Co-localization of Z_TRI_ (green) with PDGFRβ (red) in normal and HCC clinical specimens. The nuclei of cells were visualized by DAPI staining (blue)

A single chain of Z_TRI_ consists of an affibody domain against PDGFRβ (7.5 K_D_) and a self-trimerizing domain (8 K_D_) at its C terminus. Z_TRI_ affibody was recombinantly expressed in *E. coli* M15 and purified by Ni–NTA affinity chromatography. Approximately 30 mg Z_TRI_ can be obtained from 1 l of lysogeny broth and all proteins exist in soluble form. The molecular weight of Z_TRI_ was estimated at 15 K_D_ by SDS-PAGE under reduced conditions. However, SEC revealed that the molecular weight of Z_TRI_ was approximately 45 K_D_ under natural conditions, indicating that Z_TRI_ existed as a trimer in solutions (Supplementary Material Fig. S1). FAM-labeled Z_TRI_ also showed specific binding to PDGFRβ^+^ pericytes (Fig. 2a). Subsequent biolayer interferometry indicated that the affinity (K_D_) of Z_TRI_ for PDGFRβ-Fc was approximately 0.468 nM, which was much lower than 24.96 nM of Z_MON_ (Fig. 2b). Moreover, the immunofluorescent assays showed that FAM-labeled Z_TRI_ was greatly accumulated in carcinoma specimen and co-localized well with PDGFRβ in HCC tissues (Fig. 2c), suggesting that Z_TRI_-based tracer might be developed for detection of PDGFRβ overexpressed in HCC.

### *Preparation and characteristics of [*^*68*^*Ga]Ga-DOTA-ZTRI*

**Fig. 3:**
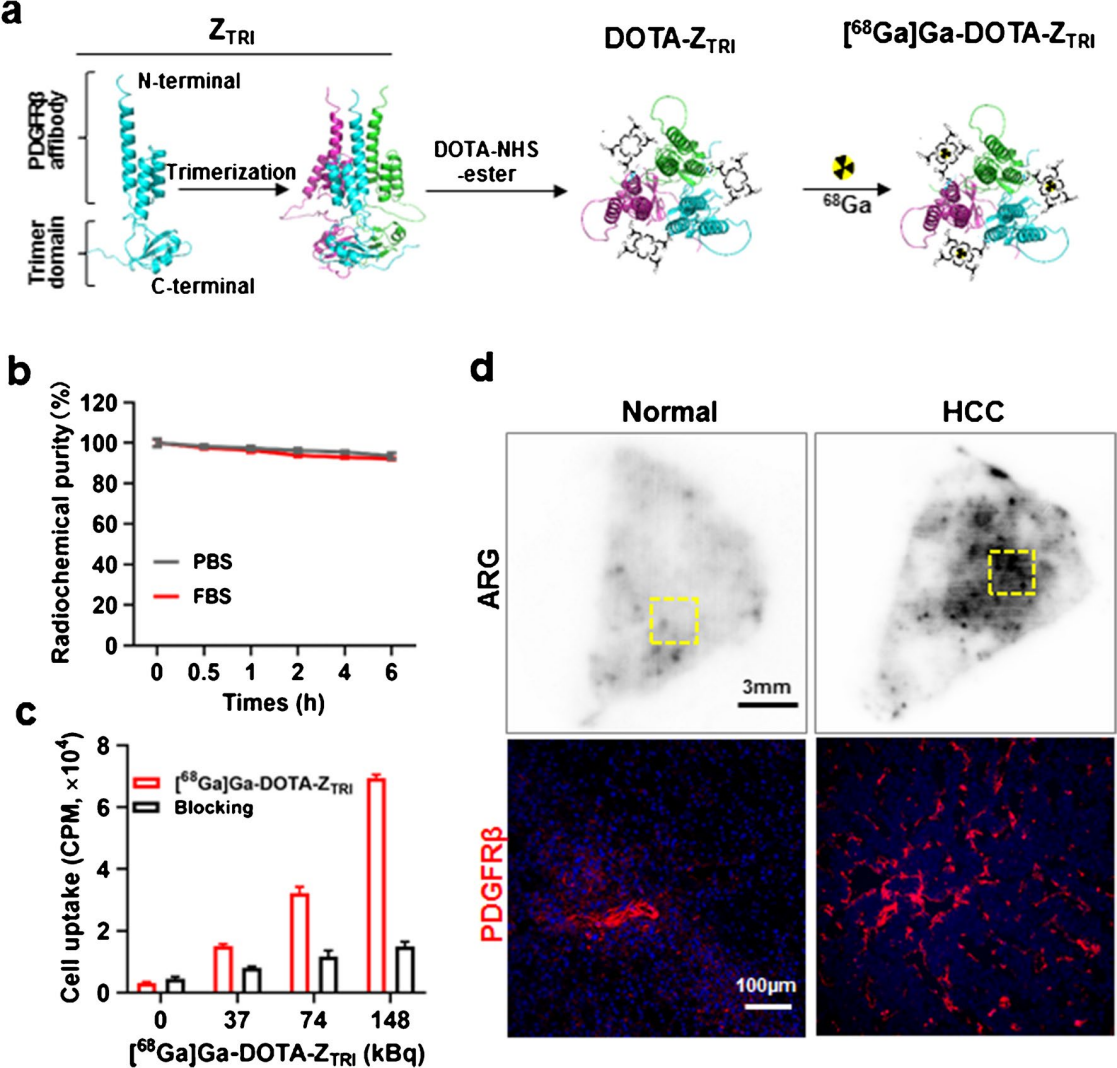
**a** Schematic illustration for the preparation of [^68^Ga]Ga-DOTA-Z_TRI_. Z_TRI_ affibody was conjugated with DOTA-NHS ester followed by radiolabeling with ^68^Ga. **b** Stability of [^68^Ga]Ga-DOTA-Z_TRI_ in PBS and FBS for 6 h in vitro. **c** Cellular binding assay of [^68^Ga]Ga-DOTA-Z_TRI_ to PDGFRβ^+^ pericytes. **d** Autoradiography of [^68^Ga]Ga-DOTA-Z_TRI_ and PDGFRβ immunofluorescent staining in human liver tissues with or without HCC

The schematic of radiolabeling procedure is shown in Fig. 3a. Briefly, bifunctional chelator DOTA-NHS was conjugated to Z_TRI_ for further radiolabeling. The peak of 15,144 Da in mass spectrometry evaluation was consistent with the single chain of Z_TRI_ while the peak of 15,531 Da was identified as the DOTA-Z_TRI_ conjugate (Supplementary Material Fig. S2a). The radiochemical yield of [^68^Ga]Ga-DOTA-Z_TRI_ was approximately 96.3 ± 2.4% (*n* = 5) under the defined conditions (Supplementary Material Fig. S2b), and the product showed satisfactory stability in fetal bovine serum (FBS) throughout 6 h (Fig. 3b). Subsequently, cell binding assays showed a dose-dependent uptake of [^68^Ga]Ga-DOTA-Z_TRI_ to PDGFRβ^+^ pericytes, while pre-incubation with 250-fold excess of unlabeled Z_TRI_ dramatically blocked the radioactive signal (Fig. 3c), indicating that the cellular uptake of [^68^Ga]Ga-DOTA-Z_TRI_ was PDGFRβ dependent. Figure 3d shows that PDGFRβ^+^ cells in HCC tissues were more than that in normal liver tissues. Accordingly, autoradiography demonstrated that rich [^68^Ga]Ga-DOTA-Z_TRI_ was accumulated in HCC tissues but not in normal liver tissues, indicating that [^68^Ga]Ga-DOTA-Z_TRI_ showed Z_TRI_-dependent binding to PDGFRβ-expressing cells.

### *Biodistribution and PET imaging feasibility of [*^*68*^*Ga]Ga-DOTA-ZTRI*

**Fig. 4:**
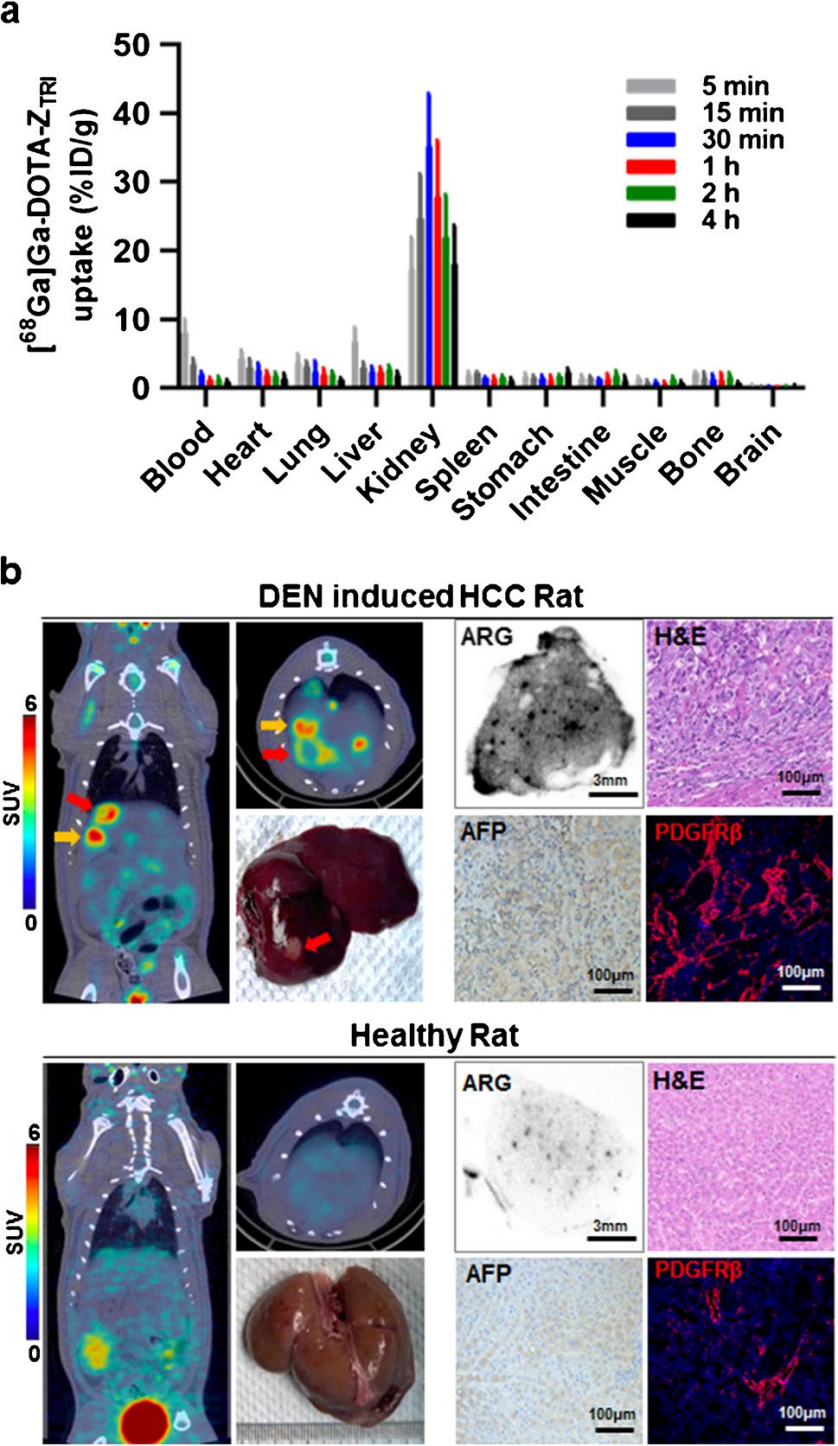
**a** Biodistribution of [^68^Ga]Ga-DOTA-Z_TRI_ in BALB/c mice. Mice were intravenously injected with 740 kBq of [^68^Ga]Ga-DOTA-Z_TRI_ followed by collection the majority organs at 5 min, 15 min, 30 min, 1 h, 2 h, and 4 h post-injection (*N* = 5). Red arrow points out the necrotic lesion and the yellow arrow points out the nodule lesion. **b** PET/CT imaging of [^68^Ga]Ga-DOTA-Z_TRI_ in DEN-induced primary HCC rats and healthy ones. Rats were intravenously injected with 5.55 MBq of [^68^Ga]Ga-DOTA-Z_TRI_ and scanned at 1 h post-injection. Tumor lesion was pointed out by red arrows. The corresponding liver tissues were collected and [^68^Ga]Ga-DOTA-Z_TRI_ uptake was investigated by autoradiography after scanning. H&E staining was used to identify the structure of tumor tissues, while AFP immunohistochemical staining and PDGFRβ immunofluorescent staining were performed as index of HCC

Biodistribution of [^68^Ga]Ga-DOTA-Z_TRI_ demonstrated no significant non-specific uptake in major organs and rapid excretion through the kidney–bladder system (Fig. 4a). Notably, although a relative high uptake (6.74 ± 2.11%ID/g) of [^68^Ga]Ga-DOTA-Z_TRI_ was measured in livers at 5 min post-injection, it decreased rapidly within 0.5 h, which is conducive for PET imaging.

To evaluate the PET imaging feasibility of [^68^Ga]Ga-DOTA-Z_TRI_, rats with DEN-induced primary HCC were administered with [^68^Ga]Ga-DOTA-Z_TRI_ and received PET scanning at 1 h post-injection. As shown in Fig. 4b, the radioactivity in normal livers were evenly low (SUVmax 1.04), whereas significant focal signals (SUVmax 5.77) were observed in DEN-induced rat liver. The lesion labeled by the red arrows was a necrotic lesion indicated in the liver photo, while the hot spot (yellow arrow) underneath is another nodule lesion within the right lobe of liver, which is not able to be observed on the surface photo but confirmed by dissection. Accordingly, the autoradiography intensity of [^68^Ga]Ga-DOTA-Z_TRI_ in HCC tissues was greater than that in normal liver tissues. In fact, abnormal hepatocytes were observed in liver tissue derived from rat with primary HCC, which was accompanied by overexpression of alpha-fetoprotein (AFP) and PDGFRβ. These results demonstrated that the liver uptake of [^68^Ga]Ga-DOTA-Z_TRI_ was closely correlated to the number of PDGFRβ-expressing cells.

**Fig. 5:**
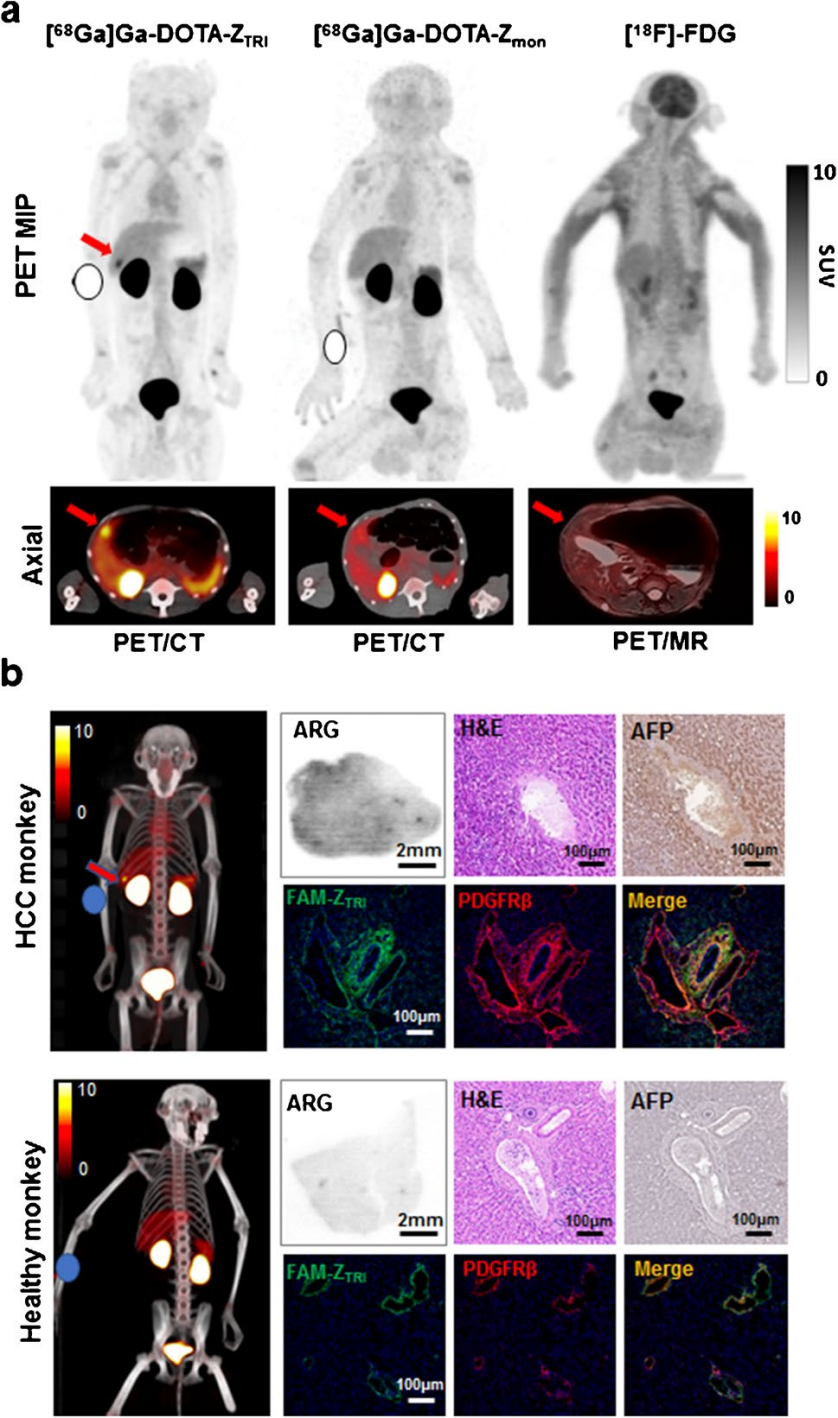
PET/CT imaging of [^68^Ga]Ga-DOTA-Z_TRI_ in rhesus monkeys. **a** Representative PET imaging of rhesus monkey with primary HCC. Monkeys were intravenously injected with a dose of 3.7 MBq/kg of [^68^Ga]Ga-DOTA-Z_TRI_, [^68^Ga]Ga-DOTA-Z_MON_, or [^18^F]-FDG, respectively. Tumor lesion is indicated by red arrows. **b** Paired comparison of HCC monkey and healthy monkey. Uptake of [^68^Ga]Ga-DOTA-Z_TRI_ in liver and tumor were evaluated using fused PET/CT imaging and autoradiography. H&E staining and AFP immunohistochemical staining were performed to identify whether the tissue is cancerous. Co-localization of Z_TRI_ (green) with PDGFRβ (red) in collected liver tissues was verified by immunofluorescent staining. The nuclei of cells were visualized by DAPI staining (blue)

To further evaluate the potential of [^68^Ga]Ga-DOTA-Z_TRI_ in clinical translation, we investigated the reactivity of [^68^Ga]Ga-DOTA-Z_TRI_ in non-human primates. Rhesus monkeys were administered with [^68^Ga]Ga-DOTA-Z_TRI_ with a dose of 3.7 MBq/kg followed by PET/CT scanning at 1 h post-injection. It was found that the tracer [^68^Ga]Ga-DOTA-Z_TRI_ was rapidly cleaned from major organs and excreted by kidneys and bladders, which is similar to that in rodent animals. The SUV value in the normal livers ranged from 1.02 to 1.67. Interestingly, a 6.95 × 4.4-mm lesion was indicated by [^68^Ga]Ga-DOTA-Z_TRI_ at the inferior segment of right lobe of the liver in one monkey (Fig. 5a). Strong radioactivity (SUVmax of 7.2) of [^68^Ga]Ga-DOTA-Z_TRI_ was measured in this lesion compared to mild uptake (SUVmax 2.36) of [^18^F]-FDG and moderate uptake (SUVmax 4.14) of [^68^Ga]Ga-DOTA-Z_MON_ affibody. Cancer cells and overexpression of AFP in the tissues derived from this lesion were indicated by H&E and AFP stanning, indicating that this monkey was a rare case of naturally idiopathic hepatic carcinoma. In addition, Z_TRI_ was well colocalized with PDGFRβ overexpressed cells in these tissues (Fig. 5b), indicating that the accumulation of [^68^Ga]Ga-DOTA-Z_TRI_ in the liver with HCC was associated to PDGFRβ-expressing cells. Absorbed dose across all subjects are shown in Supplementary Material Table S1, and the calculated effective dose was 2.03E-02 mSv/mBq based on the primate models. These results demonstrated that [^68^Ga]Ga-DOTA-Z_TRI_ has potential as PET tracer for clinical translation in HCC detection.

## Discussion

HCC is a malignant carcinoma with extremely high mortality, which is attributed to the majority of patients who are diagnosed at advanced stages. Ultrasonography, CT, and magnetic resonance imaging (MRI) are valuable techniques for the diagnosis of HCC. However, these methods are of limited value in detection of HCC with small sizes (< 2 cm) at the early stages (27). [^18^F]-FDG PET is helpful for detecting extrahepatic metastases (28), but it is also limited by reduced uptake of FDG by tumor cells, which is not ideal for early detection of primary HCC lesions. In this study, we developed a novel [^68^Ga]Ga-radiolabeled affibody conjugate by targeting PDGFRβ on tumor vessels, and validated the potential for this conjugate as an alternative PET tracer for visualizing primary HCC tumor lesions in both rats and rhesus monkeys.

Angiogenesis is a representative character throughout the occurrence and progression of hepatocellular carcinoma. A series of angiogenesis inhibitors, such as bevacizumab, sorafenib, lenvatinib, and donafenib, have been developed as drugs for HCC treatment (29, 31), suggesting that tumor vascular-targeting molecular imaging might be promising for HCC diagnosis. In fact, a series of vascular targeted targets have been identified, such as CD146 (32). Tumor vessels predominantly consist of endothelial cells and pericytes from inner to outside. Notably, due to the irregular arrangement of endothelial cells, pericytes are exposed to tracers in the blood. However, in normal blood vessels, pericytes are covered by the intact endothelium, which prevents the visualization of normal vessels by targeting pericytes (17). Thus, targeting the distinguished biomarker of pericytes becomes an attractive strategy for tumor imaging. PDGFRβ is the representative biomarker of tumor vessel–associated pericytes (33), and we also verified the good colocalization of PDGFRβ and blood vessel biomarker CD31 in the HCC tissues of monkey (Supplementary Material Fig. S3), indicating that PDGFRβ is a potential target for molecular imaging of vascularized tumors.

The pericyte-targeting tracer developed in this study is based on a trimeric affibody against PDGFRβ. Affibody molecules is a class of versatile non-immunoglobulin affinity proteins. Their small size (6.5–7 K_D_) and robust stability make them attractive for multifunctional modification and radiolabeling. Due to the high affinity and rapidly clearance, recently, several affibody-based tracers targeting human epidermal growth factor receptor type 2 (HER2) (34, 35) had been evaluated in both preclinical and clinical studies. In our previous studies, we have successfully prepared the monomeric and dimeric affibodies against PDGFRβ (21, 36). Compared to those affibody conjugates, this trimeric Z_TRI_ takes advantage of improved affinity, which triggered our interest on evaluating its potential as tracer for HCC diagnosis. However, the increased molecular mass and more complex spatial structure induced less tolerance to temperature through radiolabeling process. [^68^Ga]Ga-DOTA-Z_TRI_ conjugates indicated obvious sediment above 80 °C in radiolabeling process, while unsatisfactory product yield would be obtained under low temperatures below 40 °C.

Biodistribution assay demonstrated that the [^68^Ga]Ga-DOTA-Z_TRI_ was predominantly excreted by urinary systems. Unlike the monomeric affibody with low liver uptake, a relatively higher liver uptake (6.74 ± 2.11% ID/g) of [^68^Ga]Ga-DOTA-Z_TRI_ was observed in the initial 5 min, which might be caused by the increased molecular mass of protein. Nevertheless, the tracer was rapidly cleared from liver within 0.5 h, which would not interfere with tumor imaging. Interestingly, although PDGFRβ is an exclusive biomarker of pericytes with low expression in normal tissues, activated hepatic stellate cell would also express PDGFRβ during inflammatory repair, which should be noted to differentiate from malignancies (37). To evaluate the acute liver and kidney toxicity, mice were intravenously injected with 50 MBq of [^68^Ga]Ga-DOTA-Z_TRI_ or equal moles of Z_TRI_ (50 µg), and mice treated with PBS were considered as the control group (*n* = 5). Three groups did not show significant difference of body weights during continuous observation up to 7 days, and no obvious pathological changes were found in livers and kidneys (Supplementary Material Fig. S4). Moreover, strong radioactivity signal was observed in kidneys in PET images, which seems to have a negative impact for clinical application. Nevertheless, similar high accumulation was found in other studies of [^68^Ga]Ga-NOTA-MAL-Cys-MZHER_2:342_ and [^68^Ga]Ga-ABY-025 in mice models, while further clinical data indicated effective evacuation in these organs in several hours (38, 39). In fact, drinking plenty of water and diuretic operation would ensure effective evacuation of [^68^Ga]Ga-DOTA-Z_TRI_ from kidneys in 3 h post-injection in our ongoing unpublished clinical trial. Thus, the absorbed dose to the bladder wall is significantly lower than investigated. This suggests that high renal accumulation of [^68^Ga]Ga-DOTA-Z_TRI_ observed in animal models may not indicate adverse effect on clinical imaging. In addition to diagnosis, due to the smaller thickness of tumor blood vessels, Z_TRI_ might also serve as a carrier for the beta radionuclide (lutecium-177 or yttrium-90) and alpha particles (such as actinium-225 or astatine-211) with short path for target radionuclide therapy in the future.

## Conclusion

The PDGFRβ-targeted trimeric affibody conjugate [^68^Ga]Ga-DOTA-Z_TRI_ has a highly specific binding affinity to tumor blood vessels of hepatocellular carcinoma. Our results demonstrated that PDGFRβ-targeted PET imaging is a very promising modality with clinically translative value for diagnosis of HCC.

## Supplementary information

Below is the link to the electronic supplementary material.

Supplementary file1 (TIF 945 KB)

Supplementary file2 (TIF 963 KB)

Supplementary file3 (TIF 824 KB)

Supplementary file4 (TIF 1389 KB)

Supplementary file5 (PNG 42 KB)
