# Supplementary figures and images for: Preclinical evaluation of 68Ga-radiolabeled trimeric affibody for PDGFRβ-targeting PET imaging of hepatocellular carcinoma
Source: Eur J Nucl Med Mol Imaging. 2023 May 31;50(10):2952–61. doi: 10.1007/s00259-023-06260-x (PMC10382327; doi:10.1007/s00259-023-06260-x)

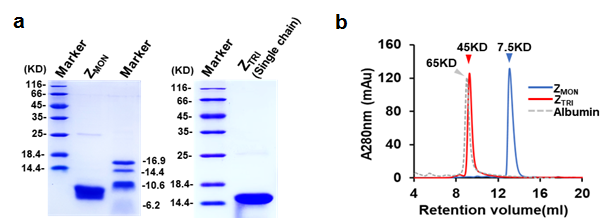

Supplement: Supplementary file 1 — Supplementary file1 (TIF 945 KB) [file 259_2023_6260_MOESM1_ESM.tif]

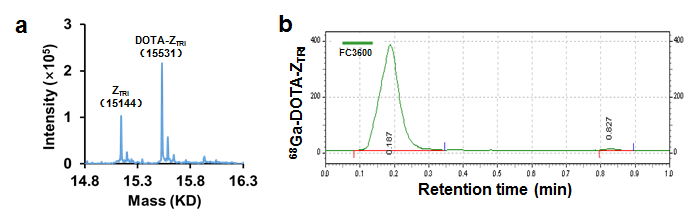

Supplement: Supplementary file 2 — Supplementary file2 (TIF 963 KB) [file 259_2023_6260_MOESM2_ESM.tif]

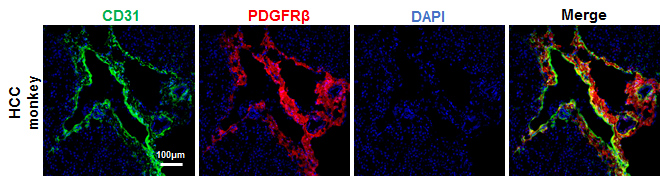

Supplement: Supplementary file 3 — Supplementary file3 (TIF 824 KB) [file 259_2023_6260_MOESM3_ESM.tif]

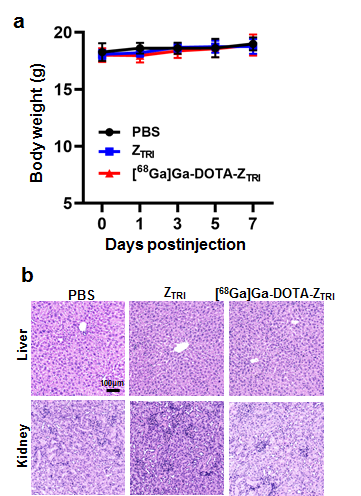

Supplement: Supplementary file 4 — Supplementary file4 (TIF 1389 KB) [file 259_2023_6260_MOESM4_ESM.tif]

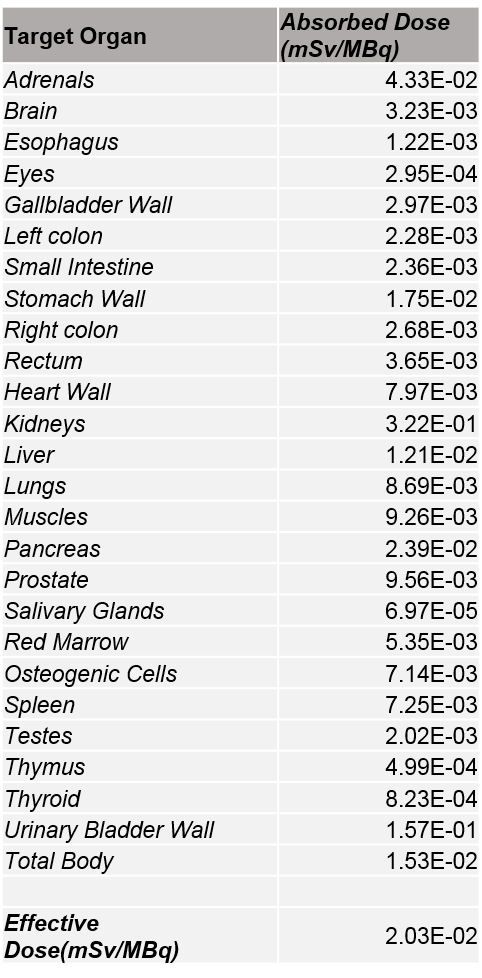

Supplement: Supplementary file 5 — Supplementary file5 (PNG 42 KB) [file 259_2023_6260_MOESM5_ESM.png]
